# The Impact of Tumor Heterogeneity on Diagnostics and Novel Therapeutic Strategies in Multiple Myeloma

**DOI:** 10.3390/ijms20051248

**Published:** 2019-03-12

**Authors:** Leo Rasche, K. Martin Kortüm, Marc S. Raab, Niels Weinhold

**Affiliations:** 1 Department of Internal Medicine 2, University Hospital of Würzburg, 97080 Würzburg, Germany; Kortuem_M@ukw.de; 2 Myeloma Center, University of Arkansas for Medical Sciences, Little Rock, AR 72205, USA; 3 Department of Internal Medicine V, University Hospital of Heidelberg, 69120 Heidelberg, Germany; Marc.Raab@med.uni-heidelberg.de (M.S.R.); Niels.Weinhold@med.uni-heidelberg.de (N.W.)

**Keywords:** multiple myeloma, spatial heterogeneity, risk stratification, minimal residual disease, targeted therapy, clinical imaging, immunotherapy, daratumumab

## Abstract

Myeloma is characterized by extensive inter-patient genomic heterogeneity due to multiple different initiating events. A recent multi-region sequencing study demonstrated spatial differences, with progression events, such as *TP53* mutations, frequently being restricted to focal lesions. In this review article, we describe the clinical impact of these two types of tumor heterogeneity. Target mutations are often dominant at one site but absent at other sites, which poses a significant challenge to personalized therapy in myeloma. The same holds true for high-risk subclones, which can be locally restricted, and as such not detectable at the iliac crest, which is the usual sampling site. Imaging can improve current risk classifiers and monitoring of residual disease, but does not allow for deciphering the molecular characteristics of tumor clones. In the era of novel immunotherapies, the clinical impact of heterogeneity certainly needs to be re-defined. Yet, preliminary observations indicate an ongoing impact of spatial heterogeneity on the efficacy of monoclonal antibodies. In conclusion, we recommend combining molecular tests with imaging to improve risk prediction and monitoring of residual disease. Overcoming intra-tumor heterogeneity is the prerequisite for curing myeloma. Novel immunotherapies are promising but research addressing their impact on the spatial clonal architecture is highly warranted.

## 1. Introduction

Multiple myeloma (MM) is a highly heterogeneous disease of clonal plasma cells (PC), which accumulate in the bone marrow (BM). While some MM patients suffer from extensive bone disease with presence of multiple osteolytic lesions, other patients have cytopenias as leading clinical problem, caused by an obliterative BMPC infiltration and other factors [1]. One-third of newly diagnosed (ND) MM patients present with renal failure due to cast nephropathy, amyloidosis, light or heavy chain deposition disease, and/or hypercalcemia [2]. Extramedullary disease (EMD) may be present, as well as elevated counts for circulating PCs, with <5% of NDMM patients having plasma cell leukemia [3]. Patients with multiple osteolytic lesions according to computer tomography (CT) scans may have a low BMPC infiltration at the iliac crest site. This pattern, which is called macrofocal disease, is seen in 6% of NDMM patients [4].

Survival outcomes reflect disease heterogeneity in MM. On the one hand, MM patients with a progression-free survival of more than 15 years, who can be considered to be “functionally” cured, have been observed [5]. On the other hand, a subgroup of ≈15% of patients experience dismal outcomes with a median survival of less than 2 years [6]. In the last decade, the molecular makeup of MM has been extensively investigated and inter-patient and intra-tumor heterogeneity have been identified as the major causes underlying the heterogeneous clinical presentation [7]. In this review article, we aim to describe the impact of tumor heterogeneity on treatment strategies. Therefore, we divided the manuscript into three parts. First, we briefly introduce the concept of inter-patient and intra-tumor heterogeneity in the context of myeloma genomes. In the second section, we describe the impact of different types of tumor heterogeneity on novel treatment strategies such as risk-adapted or minimal residual disease triggered therapy, and finally we describe diagnostic and treatment approaches that can be applied to account for inter-patient and intra-tumor genomic heterogeneity.

## 2. Inter-Patient Versus Intra-Tumor Heterogeneity

An established approach to discussing heterogeneity at the molecular level is to distinguish between: (1) inter-patient heterogeneity, which refers to variation of tumor features between patients; and (2) intra-tumor heterogeneity, which is characterized by molecular differences within one tumor [8]. MM is an ideal example for discussing these two levels of heterogeneity as its complex genomic makeup has been extensively investigated in recent years.

### 2.1. Inter-Patient Tumor Heterogeneity

**Figure 1:**
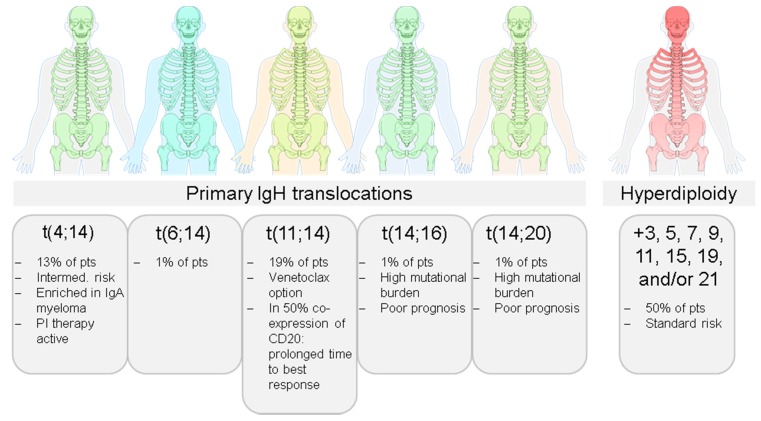
Inter-patient heterogeneity in Multiple Myeloma. The two main pathogenetic groups hyperdiploid and non-hyperdiploid can be distinguished in myeloma. However, there are multiple different initiating events at the chromosomal level, resulting in a high level of inter-patient heterogeneity in this disease, which is also reflected in heterogeneous treatment responses and outcomes.

Cytogenetic analyses have shown that MM is not a single disease but presents with unique features at the molecular level in each patient. Yet, two main pathogenetic groups can be distinguished based on primary, also referred to as initiating, chromosomal events. While hyperdiploid (HD) MM presents with multiple trisomies of the odd-numbered chromosomes 3, 5, 7, 9, 11, 15, 19, and 21, where non-hyperdiploid (NHD) MM is associated with primary immunoglobulin heavy-chain (IgH) translocations, such as t(4;14), t(11;14), t(14;16), or t(14;20), which result in overexpression of specific oncogenes located on the respective translocation partner chromosome [9] (Figure 1). Early evolution of MM is also driven by inherited variation [10], and even in this context, inter-patient heterogeneity can be seen. For example, the association between the CCND1 c.870G>A single nucleotide polymorphism and myeloma risk is restricted to patients with a primary t(11;14) IgH translocation, which leads to overexpression of *CCND1* [11].

During further disease evolution, myeloma cells usually acquire additional chromosomal aberrations, which eventually result in increased fitness, the so called secondary or progression events [12]. These include deletion of the short arm or gain of the long arm of chromosome 1 (del(1p) and gain(1q), respectively); deletion of the short arm of chromosome 17 (del(17p)), which includes the tumor-suppressor gene *TP53*; or translocations involving the *MYC* locus on chromosome 8. According to recent sequencing efforts, *NRAS*, *KRAS*, and *TP53* mutations are the main drivers of myeloma evolution at the single nucleotide level, resulting in an additional level of complexity [13,14,15,16]. Notably, certain driver gene mutations seem to be enriched in specific molecular subgroups, e.g., *NRAS* mutations affecting the Q61 codon are more frequently found in HD and t(11;14) myeloma compared to other subgroups [17].

Using tumor initiating events to better understand the complex global gene expression profiles (GEP) of myeloma cells, Bergsagel and colleagues developed the so-called TC classification [18]. It is based on the expression of D-type cyclins and the type of IgH translocation, including the groups 11q, 6p, MAF, 4p, D1, D1 + D2, D2, and none. Another attempt to classify MM using GEP was published by the University of Arkansas for Medical Sciences (UAMS) myeloma team [19]. The UAMS molecular classification is based on unsupervised clustering of expression data and recognizes seven different molecular subgroups. The HY group contains HD cases. The CD-1 and CD-2 groups include patients with translocations t(11;14) or t(6;14). The CD-2 group differs from the CD-1 by the expression of the early B-cell markers CD20 and PAX5. Upregulation of FGFR3 and/or MMSET defines the MS group, while the MF group is characterized by over-expression of c-MAF or MAFB. A low number of bone lesions is seen in the low bone disease (LB) group, and the proliferation (PR) group is associated with high expression of proliferation related genes.

An important step in elucidating inter-patient molecular heterogeneity of MM was the development of GEP-based risk predictors, which allows for assigning patients to “high” or “low” risk categories. The UAMS GEP70 risk score is based on the ratio of the mean expression level of up- to down-regulated genes among 70 genes linked to early disease-related death [20]. Most up-regulated genes are located on the long arm of chromosome 1, and many down-regulated genes map to the short arm of this chromosome 1. The predictor has a high specificity for identification of patients with poor event-free and overall survival, constituting 10–15% of NDMM patients.

In summary, MM is a complex disease with extensive inter-patient heterogeneity due to multiple different initiating and progression events at the chromosomal and single nucleotide level, which is also reflected at the gene expression level.

### 2.2. Intra-Tumor Heterogeneity

**Figure 2:**
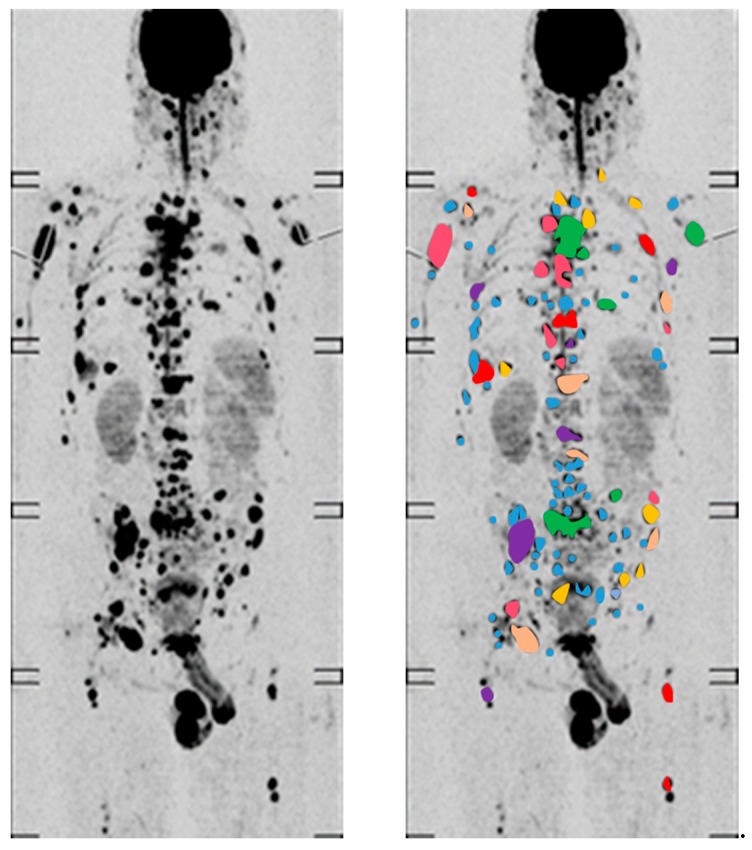
Intra-tumor heterogeneity in Multiple Myeloma. According to recent multi-region sequencing studies spatial genomic heterogeneity is a common phenomenon in myeloma. Tumor driver mutations and high-risk genomic aberrations can be restricted to one focal lesion and absent at other FLs or the iliac crest. Thus, an imaging finding with multiple FLs strongly suggests extensive intra-tumor heterogeneity.

Using next generation sequencing and performing single-cell genetic analyses, Melchor et al. identified two to six different major myeloma subclones at presentation [21]. They observed clonal extinction and the emergence of new clones that acquire additional mutations during treatment, supporting a Darwinian model of evolution in myeloma. According to this model, new mutations can result in better adaptation and outgrowth of clones, outcompeting previously dominant tumor clones [12,22]. Since MM primarily grows in the BM, free movement of tumor cells leading to a rapid and homogenous dissemination of clones was assumed until recently. However, branching evolution has been identified as one of the main patterns in longitudinal molecular studies of MM [13,23,24,25,26]. Branching evolution during treatment, where multiple clones emerge from a common ancestor and different clones dominate at the diagnosis of MM and at relapse, instead suggest the pre-existence of drug-resistant clones. Indeed, using multi-region sequencing including focal lesions (FLs), we recently demonstrated local differences in the clonal architecture (Figure 2). While initiating events, such as the primary IgH translocations t(4;14), t(11;14), and t(14;16), were uniformly shared among investigated sites and detectable in all tumor cells of the respective patient, progression events, such as *MYC* translocations, gain(1q), or *RAS* mutations, frequently showed spatial differences. Of note, the extent of spatial heterogeneity was different between patients, constituting another level of inter-patient heterogeneity. Extensive spatial heterogeneity, where poor prognosis mutations were found to be restricted to one site in the BM, were primarily seen in patients with large FLs [27]. Analyzing evolution patterns in these patients in more detail, one patient showed large FLs that shared genomic aberrations that were not found at the iliac crest, indicating a metastatic type of evolution. In another patient, each investigated FL presented with site-unique mutations, which affected driver genes such as *BRAF* or *KRAS*. In a third patient, two spatially separated subclones and an independent minor subclone that infiltrated all investigated sites were seen, demonstrating the complex processes in the BM cavity that underlie the evolution of MM [27].

Together, MM is characterized by spatial differences in the clonal architecture, with progression events frequently being restricted to one site in the BM.

## 3. Impact of Heterogeneity on Treatment Strategies

### 3.1. Targeted Therapy

Up to 50% of NDMM patients present with mutations in *NRAS*, *KRAS*, or *BRAF*, making the MAPK pathway a good candidate for targeted therapy [28,29]. Pilot studies reported a successful use of vemurafenib in patients with BRAF V600E mutations [30,31], and complete responses were observed in some MM patients with mutations of *KRAS*, *NRAS*, or *BRAF*, who were treated with trametinib [28]. Unfortunately, no long-term responses were seen. To make sure that all tumor cells ubiquitously express the target, only aberrations, which are detectable in all investigated tumor cells—the so-called clonal mutations—should be selected for targeted treatment. However, in MM molecular tests are based on a sample from a single site, typically the iliac crest. Since regionally restricted tumor clones are a frequent feature of MM, a dominant clone at that site could just mimic clonal mutations. As an example, in one patient, we recently found an actionable BRAF V600E mutation in all tumor cells at the iliac crest, but there was an absence of this variant in myeloma cells that grew in a large FL at the lumbar spine [27]. Furthermore, in a longitudinal study, we identified spatially divergent clonal evolution as the mechanism underlying resistance: in a patient treated with vemurafenib 3 resistant, lesions developed, which all presented with new unique site-specific *NRAS* mutation [32]. Appearance of new mutations is a frequent finding at relapse [23,33,34,35]. However, it is still unclear whether resistant disease is due to new mutations acquired during treatment and/or if minor subclones are selected by therapy. Of note, the latter would point to a level of intra-tumor heterogeneity in MM that is considerably higher than the one determined by recent multi-region sequencing studies.

In theory, targeting initiating events, such as primary IgH translocations, would overcome the issue of spatial heterogeneity. Unfortunately, a successful targeted approach, e.g., via inhibition of MMSET and FGFR3 in t(4;14) clones, has yet to be demonstrated. Alternatively, molecular characteristics, which are associated with these initiating events, could be clinically useful. One example is the anti-myeloma activity of the BCL-2 inhibitor venetoclax in t(11;14)-positive MM patients. Clones harboring this translocation show a high ratio of BCL-2 expression compared to MCL-1 and BCL-Xl, and apparently, this ratio translates into a higher treatment efficacy compared to other molecular subgroups of MM. Yet, the best activity is seen in combination with proteasome inhibitors, and we expect that it will be a long journey toward successful treatment of initiating events.

In summary, drugs directly targeting initiating events in MM are still not available. Moreover, driver gene mutations are frequently dominant at one site but absent at other sites, which poses a significant challenge to targeted therapy in MM.

### 3.2. Treatment Decisions Based on Disease Risk Status

For MM patients with a high-risk disease, even the most intensive therapies have not resulted in improved outcomes. Thus, it is crucial to develop dedicated therapies for these patients. The UAMS total therapies 4 (low risk) [36] and 5 (high risk) [37], the Deutsche Studiengruppe Multiples Myelom (DSMM) V trial [38], the German-Speaking Myeloma Multicenter Study Group (GMMG) Concept trial (NCT03104842), and the mSMART algorithm used by the Mayo Clinic [39] are examples of risk-adapted strategies. A pre-requisite for such trials is the use of effective tools to identify high-risk patients at presentation. Classifiers based on GEP data are highly specific, but immediate access to an experienced sample processing laboratory, which is mandatory to obtain reliable results [40], is not standard. Alternatively, FISH results can be used to predict risk. The primary IgH translocations t(4;14) and t(14;16) and the deletion del(17p) are commonly used prognostic markers in MM, and their recent inclusion into the revised version of the International Staging System led to a significant improvement of this classifier [41]. Yet, we recently analyzed GEP70 scores in molecular subgroups, and we demonstrated that high risk signatures are only enriched in patients with a t(4;14) or t(14;16) translocations and not linked to subgroup specific characteristics like *MMSET* or *MAF* overexpression per se [42]. As a result, patients with a t(4;14) can in fact be low risk and experience long-term progression-free survival despite presenting with a negative prognostic FISH marker [42]. In line with this observation, Thanendrarajan et al. recently showed that a combination of del(17p) and GEP risk signatures provided a more precise prediction of outcome of NDMM patients [43].

Another variable that seems to impact the predictive power of del(17p) is the status of the second *TP53* allele. According to a longitudinal sequencing study, only patients with complete inactivation of tumor suppressor genes, a so called bi-allelic event, experienced poor outcome after relapse [23], in contrast to mono-allelic events, and the same was seen in newly diagnosed patients [44]. In a recent study by the U.K. group, MM patients with bi-allelic *TP53* aberrations also demonstrated dismal survival [45]. However, in this study, patients with a mono-allelic *TP53* event also suffered from a poor outcome, highlighting that even established risk factors in MM are still controversial.

The situation becomes even more complicated when spatial genomic intra-tumor heterogeneity is considered. We recently described an MM patient with GEP70 high risk status and a bi-allelic *TP53* deletion in a focal lesion in the lumbar spine but absence of this deletion and GEP70 low risk at the iliac crest site [27]. We found this type of discrepancy in GEP risk scores in ≈10% of NDMM patients with GEP70 low risk according to the iliac crest sample, thereby nearly doubling the number of NDMM patients with GEP70 high risk [27]. Importantly, the outcome of patients with discrepant scores was similar to the outcome for cases with a homogeneous distribution of GEP70 high risk clones, illustrating that high-risk subclones negatively impact prognosis even if they are not ubiquitously distributed in NDMM patients.

Together, the type of aberration needs to be considered if chromosomal aberrations impacting tumor suppressor genes are used for risk prediction, and the performance of established prognostic markers for NDMM patients is limited by spatial genomic heterogeneity.

### 3.3. Treatment Decisions Based on the Response and the Level of Minimal Residual Disease

Deep responses to treatment are associated with a good outcome in myeloma [46,47]. As a result, one important aim of MM treatment is to achieve minimal residual disease (MRD) negative states. One strategy to achieve deep responses is to perform double autologous stem cell transplantation followed by consolidation and long-term maintenance. Vice versa, treatment breaks may be an option for MRD negative patients in order to prevent serious side effects. Yet, both inter- and intra-tumor genomic heterogeneity need to be considered when treatment decisions are based on response levels. Molecular subgroups with similar long-term outcomes can differ considerably in response kinetics. Patients with a t(11;14) translocation are an illustrative example. While the CD-1 subgroup goes rapidly into remission, a maximum response occurs significantly later in the CD-2 group [42,48]. Thus, GEP data is therapeutically important, since it allows one to differentiate between these two t(11;14)-positive groups and can be used to prevent overtreatment of the CD-2 group.

Monitoring of MRD is based on a sample from a single site at the iliac crest. As exchange between clones at spatially distinct sites is limited in myeloma and highly advanced clones grow as FLs, locally different (“mixed”) responses to treatment are expected. Indeed, relapses from MRD-negative complete response (CR) and detection of different subclones at presentation and relapse in patients strongly indicate the presence of therapy-resistant tumor cells at sites other than the iliac crest [23], and our recent observations support this concept. We performed a spatial-longitudinal study with one patient who presented with more than 100 FLs and extensive spatial heterogeneity at baseline. The patient achieved an MRD-negative CR but ultimately relapsed during the course of the disease. At relapse, we sequenced a sample from a large FL with paramedullary components and a routinely collected samples from the right iliac crest. In the large FL, we identified a clone, which shared a “KRASGly12Val” with one of the baseline clones but presented with 65 new missense mutations that were not detectable at baseline despite deep multi-region sequencing. In the iliac crest sample, we found an additional resistant clone, which was also not detectable at baseline, and presented with another site-unique clonal *KRAS* mutation [49]. This observation highlights that even in MRD-negative patients, multiple spatially separated clones can survive.

In summary, the molecular makeup and differences in the spatial clonal structure need to be considered for MRD-triggered therapy decisions.

## 4. Overcoming Tumor Heterogeneity

### 4.1. Functional Imaging to Decode Intra-Tumor Heterogeneity in MM

As described above, spatial heterogeneity significantly affects personalized treatment, risk prediction, and MRD diagnostics in MM. Since assessment of multiple skeletal sites is difficult and not feasible at every center, alternative strategies are required to account for this type of heterogeneity. The functional imaging methods ^18^fluoro-deoxyglucose positron-emission tomography-computed tomography (PET-CT) and diffusion-weighted magnetic resonance imaging with background suppression (DWIBS) have emerged as useful tools for disease staging and prognosis at baseline [50,51,52,53,54]. Combining these two modalities and multi-region tumor sequencing as a first attempt to perform “radio-genomics” in MM, we recently showed that spatial heterogeneity was positively associated with the size of biopsied FLs [27]. Site-specific progression markers, such as *RAS* mutations or inactivation of *TP53*, were primarily seen in large FLs with a diameter of >2.5 cm. Thus, we hypothesized that the FL size as a surrogate marker for intra-tumor heterogeneity negatively impacted treatment success. Supporting this hypothesis, an imaging pattern characterized by large sized FLs was associated with poor outcome even in patients who were classified as favorable according to the revised International Staging System (ISS) and GEP70 [53], highlighting that “radio-genomics” can potentially overcome the challenge that heterogeneity poses to risk prediction based on a single site. This study also showed that an established risk marker, more than three FLs at baseline, lost its predictive power when accounting for FL size, consistent with size being the more important imaging variable, and showing that a better understanding of myeloma biology can lead to improved risk prediction.

Our group and others have shown that functional whole-body imaging can be used to monitor residual disease in MM and improve conventional MRD diagnostics [49,55]. In our study, 24% of first-line MM patients presented with residual FLs in DWIBS and/or PET-CT at the onset of CR, and these lesions were associated with short progression-free survival [49]. Although DWIBS is more sensitive than PET-CT in this context, the two techniques are complementary because some residual FLs are only detectable in PET-CT [49]. Combining flow cytometry for MRD detection and functional imaging, we demonstrated that only 4 of 83 MRD-negative patients, who achieved CR during first-line treatment, still presented with residual FLs [49]. Thus, focally restricted residual disease is a rare event in MRD-negative NDMM patients in CR at a sensitivity of 1 × 10^−5^. In contrast, up to 50% of patients who achieve MRD-negative CR during salvage present with positive imaging tests [49]. This observation seems to suggest that a combination of molecular MRD tests and functional imaging is primarily useful in late stage patients. Yet, we recommend also performing functional imaging in first-line CR patients for the following reasons. First, in the small group of MRD-negative patients with residual lesions we observed early relapses, consistent with residual FLs in MRD-negative patients being prognostically relevant. Second, FLs in MRD-positive patients are associated with a particularly dismal outcome and a switch to alternative treatments should be discussed.

In addition to its power for the detection of high-risk features and locally restricted residual disease, functional imaging can also be used to guide molecular analyses. As an example, we recently investigated the phenomenon of FDG PET false-negativity, which is seen in ≈10% of MM patients [56]. Performing simultaneous assessment of DWIBS and PET-CT, together with GEP of CD138-positive plasma cells to better characterize patients with false-negative PET, we showed that low expression of the gene coding for hexokinase-2, which catalyzes the first step of glycolysis, was significantly associated with this phenomenon. Thus, the application of functional imaging revealed differences in metabolism as another level of tumor heterogeneity in MM. Nevertheless, we need to emphasize that the molecular makeup of tumor cells, which are absent at the iliac crest, cannot be deciphered with this approach. Other non-invasive approaches, such as molecular tests based on circulating tumor cells or DNA, are probably more useful in this context and first results of these studies are promising [14,57,58,59]. The same holds true for proteomic screening of plasma samples targeting proteins showing aberrant expression. These may provide an additional means of identifying heterogeneity, with potential for implementation as a biomarker approach in routine hospital laboratories [60]. However, a comprehensive longitudinal study combining multi-region sequencing including FLs and circulating cells/DNA, as well as protein profiling, has not been performed yet. Thus, the sensitivity and usefulness of the latter are still not clear.

In summary, whole body functional imaging is one way to account for the negative impact of spatial heterogeneity on current risk classifiers and detection of residual disease but does not allow for deciphering the molecular characteristics of the respective clones.

### 4.2. Immunotherapy to Target All Tumor Subclones

Only recently, novel immunotherapies have dramatically changed the treatment landscape of MM. In contrast to the complex genome, the targets for immunotherapies are considered to be more homogenous and stable, making this type of treatment very promising for eradication of all tumor subclones [61]. Importantly, the cytotoxicity of immunotherapy does not rely on the induction of apoptosis alone but leads to external lysis of the target cells by granzymes, perforins, or complement. Thus, in theory, a complex clonal architecture with spatial genomic heterogeneity should not limit the activity of immunotherapies.

Allogeneic stem cell transplantation (allo SCT) can be considered to be the prime example of immunotherapy as its activity is based on the transfer of a “new” immune system and the resulting graft-versus-myeloma effect. Yet, analyzing relapse patterns of 155 patients who underwent allo SCT for MM, we noted EMD relapses in 32% of the patients [62]. For other treatment modalities, the observed rate for EMD at relapse is considerably lower (6–15% of patients) [63,64,65,66]. The increased risk for EMD after allo SCT was also seen in other studies [67,68,69,70], supporting a concept in which allo SCT puts a specific, yet poorly understood, selective pressure on MM cells favoring extramedullary progression. In line with this assumption, EMD frequently occurred in the absence of a detectable intramedullary relapse [63].

**Figure 3:**
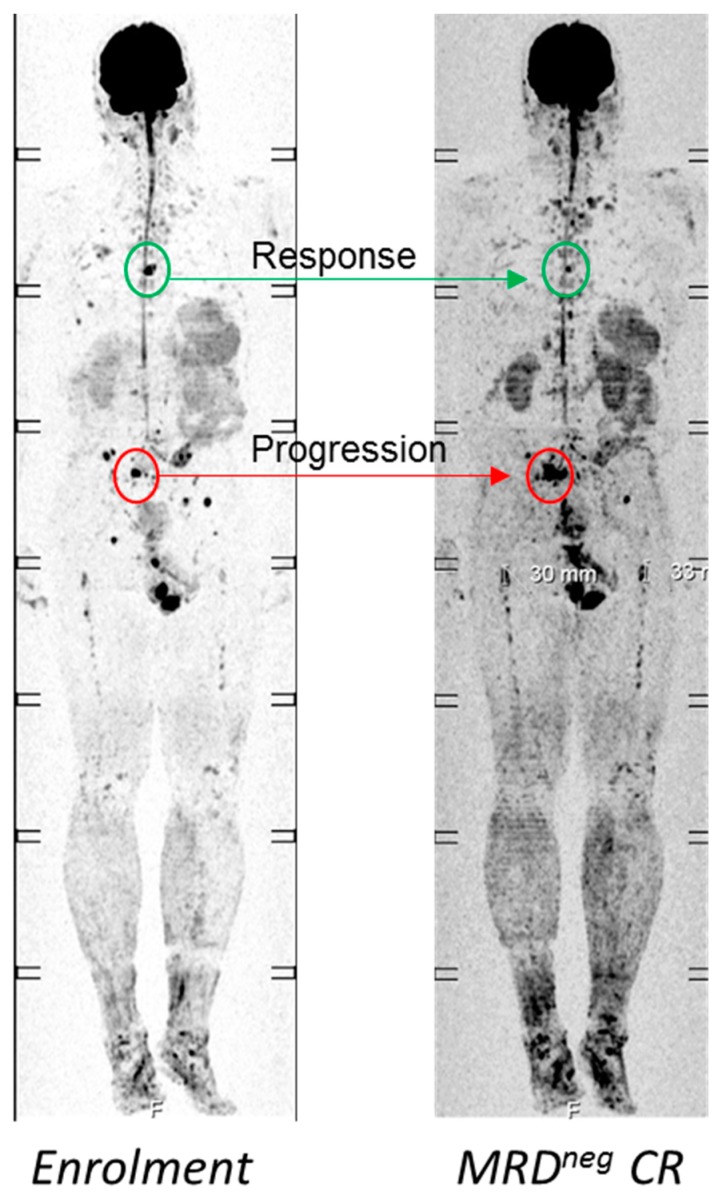
Example for a mixed response to Daratumumab. At enrolment into salvage therapy, which contained Daratumumab combined with Pomalidomide and Dexamethasone, the patient presented with an M-protein of 3 g/dl and multiple focal lesions (FLs). The patient achieved an MRD-negative stringent CR but still presented with FLs. While an FL in the thoracic spine was improved (green circle), another FL in the pelvis had increased in size (red circle), highlighting a mixed response to Daratumumab.

Interestingly, phase 3 and real-world data show a similar trend for the monoclonal anti-CD38 antibody Daratumumab, which was approved by the European Medicines Agency in 2016. Response rates are considerably lower in EMD patients compared to patients with intramedullary disease (overall response rate of just 0–10%) [71,72], suggesting that the activity of monoclonal antibodies is severely limited in EMD, too. Our own unpublished observations also indicate an ongoing and profound impact of spatial heterogeneity on the efficacy of Daratumumab, and even the presence of intra-medullary resistant lesions: some MM patients, who experienced deep responses according to BM samples from the iliac crest, still presented with active FLs in the two functional imaging modalities DWIBS or PET-CT. Furthermore, in some patients, a radiological heterogeneous response was seen, with some FLs decreasing and others progressing (Figure 3). We propose that specific tumor-intrinsic and -extrinsic features of EMD and resistant intra-medullary lesions explain these observations, but the underlying mechanisms have yet to be elucidated.

Of note, T cell engaging immunotherapies, such as chimeric antigen receptor (CAR) T cells or bispecific T cell engaging antibodies, trigger strong responses in heavily pretreated MM patients with refractory disease [61,73], suggesting that the clinical meaning of tumor heterogeneity needs to be re-defined in the era of novel immunotherapies for this disease. However, to the best of our knowledge, the impact of these agents on the spatial clonal architecture in MM is unknown.

## 5. Conclusions

MM is a complex disease, characterized by inter-patient and intra-tumor heterogeneity. Thus, we recommend combining molecular tests with functional imaging to improve risk prediction and monitoring of residual disease, which are highly impacted by spatial heterogeneity. Overcoming this type of heterogeneity is the prerequisite for a cure in MM. Novel immunotherapies, such as Car T cells, are promising but research addressing their impact on the spatial clonal architecture in MM is highly warranted.
